# Determination of starting dose of the T cell-redirecting bispecific antibody ERY974 targeting glypican-3 in first-in-human clinical trial

**DOI:** 10.1038/s41598-022-16564-x

**Published:** 2022-07-19

**Authors:** Shun-ichiro Komatsu, Yoko Kayukawa, Yoko Miyazaki, Akihisa Kaneko, Hisashi Ikegami, Takahiro Ishiguro, Mikiko Nakamura, Werner Frings, Natsuki Ono, Kiyoaki Sakata, Toshihiko Fujii, Shohei Kishishita, Takehisa Kitazawa, Mika Endo, Yuji Sano

**Affiliations:** 1 Discovery Pharmacology Department, Kamakura Research Division, Chugai Pharmaceutical Co., Ltd., 200 Kajiwara, Kamakura, Kanagawa 247-8530 Japan; 2 Translational Research Division, Chugai Pharmaceutical Co., Ltd., 1-1 Nihonbashi-Muromachi 2-Chome, Chuo-ku, Tokyo 103-8324 Japan

**Keywords:** Cancer, Drug discovery, Immunology, Oncology

## Abstract

Currently, ERY974, a humanized IgG4 bispecific T cell-redirecting antibody recognizing glypican-3 and CD3, is in phase I clinical trials. After a first-in-human clinical trial of an anti-CD28 agonist monoclonal antibody resulting in severe life-threatening adverse events, the minimal anticipated biological effect level approach has been considered for determining the first-in-human dose of high-risk drugs. Accordingly, we aimed to determine the first-in-human dose of ERY974 using both the minimal anticipated biological effect level and no observed adverse effect level approaches. In the former, we used the 10% effective concentration value from a cytotoxicity assay using the huH-1 cell line with the highest sensitivity to ERY974 to calculate the first-in-human dose of 4.9 ng/kg, at which maximum drug concentration after 4 h of intravenous ERY974 infusion was equal to the 10% effective concentration value. To determine the no observed adverse effect level, we conducted a single-dose study in cynomolgus monkeys that were intravenously infused with ERY974 (0.1, 1, and 10 μg/kg). The lowest dose of 0.1 μg/kg was determined as the no observed adverse effect level, and the first-in-human dose of 3.2 ng/kg was calculated, considering body surface area and species difference. For the phase I clinical trial, we selected 3.0 ng/kg as a starting dose, which was lower than the first-in-human dose calculated from both the no observed adverse effect level and minimal anticipated biological effect level. Combining these two methods to determine the first-in-human dose of strong immune modulators such as T cell-redirecting antibodies would be a suitable approach from safety and efficacy perspectives.

Clinical trial registration: JapicCTI-194805/NCT05022927.

## Introduction

Over the past decade, immunotherapies have played a significant role in cancer treatment, as immune checkpoint inhibitors (ICIs) have demonstrated considerable efficacy in patients^1–3^. In addition to ICIs, various types of immuno-oncology drugs have been clinically developed to treat solid tumors. T cell-redirecting antibodies (TRABs) are the most popular types of immuno-oncology drugs. TRAB, a bispecific antibody consisting of CD3-binding and cancer antigen-binding arms, exerts a strong toxicity against cancer cells via the activation of T cells^4,5^. Catumaxomab, targeting epitherial cell sdhesion molecule (EpCAM) and CD3, was the first TRAB approved for a solid tumor (i.e., EpCAM^+^ malignant ascites) by the European Medicines Agency (EMA) in 2009, but was withdrawn due to severe toxicities^6,7^. However, various TRABs have been used in clinical trials^5,8,9^.

ERY974, a TRAB that binds to Glypican 3 (GPC3) and CD3, is currently in phase I clinical trials (JapicCTI-194805/NCT05022927). This bispecific antibody has a unique structure with a common light chain for both GPC3 and CD3, and a silent Fc to prevent GPC3-independent cytotoxicity caused by antibody-dependent cellular cytotoxicity of effector cells via the Fc portion^10,11^. GPC3 is a heparan sulfate proteoglycan and oncofetal protein whose expression is observed mainly during embryogenesis, but not in adult tissues^12^. However, GPC3 is highly expressed in various cancers, especially hepatocellular carcinoma^13^. Therefore, GPC3 is an ideal antigen for TRAB and is expected to reduce the risk of adverse events in normal tissues caused by its strong cytotoxicity.

It is difficult to predict the efficacy and toxicity of immuno-oncology drugs in humans from animal study results because immunological reactions induced by immuno-oncology drugs generally vary with animal species. In preclinical studies of other TRABs, the efficacy dose calculated from the results of experiments using murine models is several hundred higher than the actual efficacy dose in patients^14–18^, probably because the murine models, in which human T cells are injected or differentiated from CD34-positive cells in immunodeficient mice, are not immunocompetent, and human immune system is not completely reconstituted. Furthermore, TGN1412, an anti-CD28 agonistic antibody, at the first-in-human (FIH) dose (e.g., 100 μg/kg) caused cytokine release syndrome (CRS), a life-threatening severe adverse event, in all six healthy volunteers^19^. However, the administration of 50 mg/kg TGN1412, more than 500-fold higher dose than the FIH dose, was well tolerated in cynomolgus and rhesus monkeys, and it caused no adverse effect^20^. Since then, the optimal FIH dose of immuno-oncology drugs has been extensively discussed. Consequently, the EMA published a guideline for strategies to identify and mitigate risks of investigational medicinal products in FIH clinical trials^21^, and the United States Food and Drug Administration (US FDA) published S9 Nonclinical Evaluation for Anticancer Pharmaceuticals finalized by the International Council for Harmonisation of Technical Requirements for Pharmaceuticals for Human Use^22^. In addition to the conventional method based on the no observed adverse effect level (NOAEL) in appropriate animal species, a method based on the minimal anticipated biological effect level (MABEL) has been considered to determine the FIH dose of highly active immuno-oncology drugs including TRABs, which might exert severe toxicities such as cytokine release syndrome (CRS)^23^. In the MABEL approach, pharmacokinetic (PK)/ pharmacodynamic (PD) data in appropriate animal species is integrated with concentration–response curve data of the most sensitive assay using human cell lines in vitro.

Here, we aimed to determine the FIH dose of ERY974 combining conventional NOAEL, and MABEL approaches. To this end, we screened for the most sensitive in vitro assay method among cytotoxicity assay, T cell activation assay, and cytokine release assay, and then calculated the MABEL using the EC_10_ values obtained in the most sensitive assay. Furthermore, we determined the NOAEL using cynomolgus monkeys in an in vivo toxicity study. By considering the FIH doses calculated using the MABEL and NOAEL approaches, we determined the FIH dose of ERY974.

## Results

### huH-1 cell line presented the highest sensitivity to ERY974

**Figure 1:**
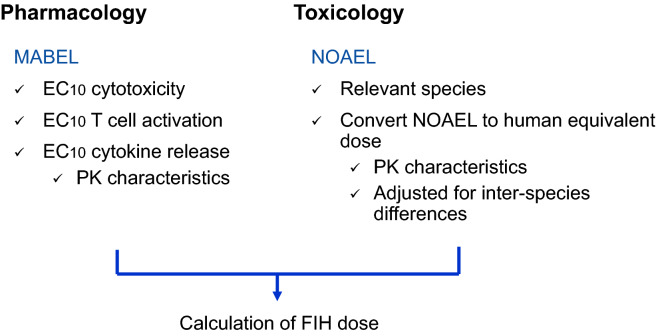
Strategy to determine the FIH dose of ERY974. Pharmacology data exploiting the MABEL approach and toxicology data in cynomolgus monkeys to determine the NOAEL were combined to determine FIH dose of ERY974. FIH, first-in-human; MABEL, minimal anticipated biological effect level; NOAEL, observed adverse effect level.

**Figure 2:**
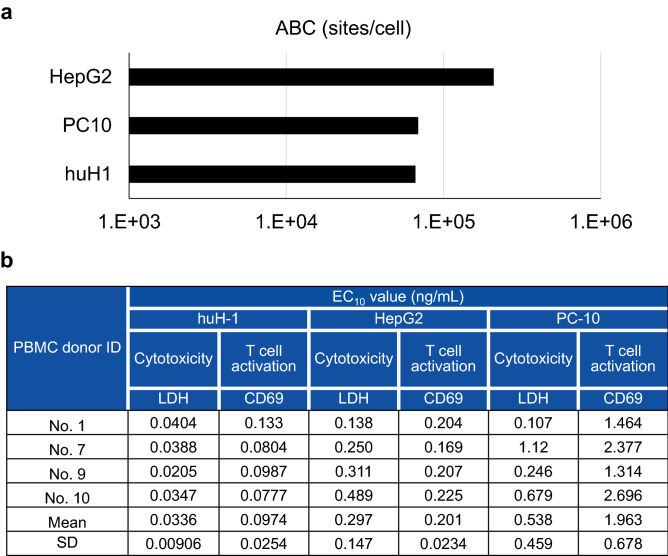
huH-1 is the most sensitive cell line to ERY974. (**a**) GPC3 expression level on the surface of HepG2, PC-10, and huH-1 cells. ABC for each cell line is shown. (**b**) The EC_10_ values of cytotoxicity and T cell activation assay of ERY974 in HepG2, PC-10, and huH-1. The PBMCs from four independent donors were used as effector cells. EC_10_ was calculated using the sigmoid Emax model. Data are shown as mean ± SD (n = 3). ABC means antigen-binding capacity.

According to the EMA and FDA guidelines, we calculated the FIH dose based on pharmacology in addition to toxicology as shown in Fig. 1. In the MABEL approach, we first screened for the cell line with the highest sensitivity to ERY974 among HepG2, PC-10, and huH-1, all of which express high level of GPC3, and therefore have been frequently used to examine the efficacy of ERY974^10^. Previously, we found that the expression level of GPC3 is an important factor influencing the sensitivity to ERY974^10^; therefore, here, GPC3 expression level on the cell surface was quantified as shown in Fig. 2a. HepG2 showed the highest GPC3 expression with more than 1 × 10^5^ of Antibody binding capacity (ABC), followed by PC-10 and huH-1. We then compared the EC_10_ values of ERY974 between the cytotoxicity assay (monitored by lactate dehydrogenase (LDH) release) and T cell activation assay (monitored by CD69 expression) in these three cell lines using Peripheral blood mononuclear cells (PBMCs) from four different donors. As shown in Fig. 2b, the mean EC_10_ value determined using the cytotoxicity assay and T cell activation assay in huH-1 cells was 0.0336, and 0.0974 ng/mL, respectively. In both assays, the EC_10_ value of huH1 is the lowest among the three cell lines, demonstrating that this was the most sensitive cell line.

### Cytotoxicity assay is the most sensitive method, and EC_10_ value in huH1 was used to calculate the FIH dose of ERY974

**Figure 3:**
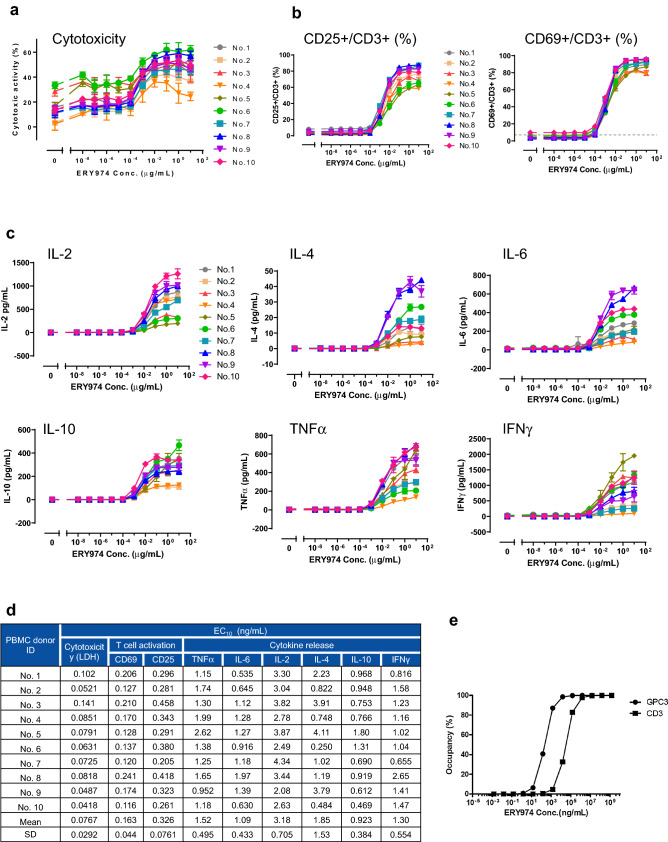
Various in vitro assays to observe the pharmacological effect of ERY974 using huH-1 and PBMCs. (**a)** Cytotoxicity assay for ERY974 in huH-1. The cytotoxicity was calculated by measuring LDH release from huH-1. (**b**) Induction of CD25 and CD69 expression in T cells treated with ERY974. T cell activation by ERY974 is measured using cytometric analysis for T cell activation markers CD25 and CD69. (**c**) Production of IL-2, IL-4, IL-6, IL-10, TNFα, and IFNγ induced by ERY974. The level of each cytokine was measured using the cytometric analysis. In all assays, PBMCs from 10 independent donors were used as effector cells, and huH-1 was used as the target cell line. Data are shown as mean ± SD (n = 3). (**d**) Cytotoxicity assay is the most sensitive assay of ERY974 in huH-1. The mean EC_10_ values of cytotoxicity, T cell activation, and cytokine production of ERY974 using the 10 PBMC samples from different donors are shown. The sigmoid Emax model was used to calculate EC_10_ for each in vitro assay. More precisely, estimated values of EC_50_, Emax and Hill coefficient were used for the calculation of EC_10_ where each pharmacological activity reaches 10% of maximum. (**e**) The RO of ERY974 to GPC3 and CD3 antigens. The RO was calculated using the following formula: RO (%) = [mAb]/([*K*_D_] + [mAb]) × 100. RO, receptor occupancy.

Next, we screened for the most sensitive assay among cytotoxicity assay, T cell activation assay (monitored by CD69 and CD25 expression), and cytokine production [measured for IL-2, IL-4, IL-6, IL-10, tumor necrosis factor α (TNFα), and Interferon γ (IFNγ)] at 24 h after ERY974 treatment (Fig. 3a-c). To consider individual differences among PBMC lots, we used randomly selected 10 PBMC samples from different donors. The calculated mean EC_10_ values in each assay are summarized in Fig. 3d. The EC_10_ value obtained in the cytotoxicity assay was 0.0767 ng/mL, which was the smallest value among the three assays, demonstrating that the cytotoxicity assay was the most sensitive assay.

### Receptor occupancy (RO) of ERY974 on GPC3 and CD3 antigens is not used for FIH dose calculation

RO can predict the pharmacodynamic effect of antibody drugs. As shown in Fig. 3e, 24 and 3348 ng/mL ERY974 was required to cover 10% of GPC3 and CD3 on the cell surface, respectively. These findings demonstrate that a high amount of ERY974 is required to achieve 10% RO. Therefore, we decided not to use RO to calculate the FIH dose of ERY974 in humans.

### Cynomolgus monkey is an appropriate species to examine the safety profiles of ERY974

**Figure 4:**
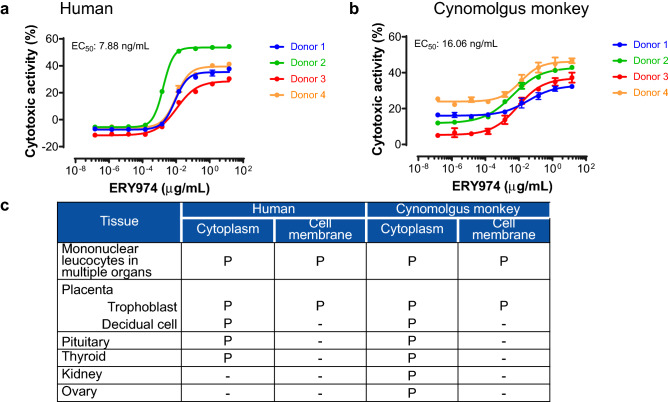
Cynomolgus monkey is a suitable species to study the safety of ERY974. (**a**) Cytotoxicity assay for ERY974 using human PBMCs. (**b**) Cytotoxicity assay for ERY974 using cynomolgus monkey PBMCs. PBMCs from four independent donors are used as effector cells, and SK-pca13a was used as the target cell. Data are shown as mean ± SD (n = 3). (**c**) Summary of tissue cross-reactivity of ERY974 in human and cynomolgus monkeys. Frozen sections of various normal tissues from human and cynomolgus monkeys were stained with biotinylated ERY974. Tissues positive for ERY974 staining are shown with “P,” and tissues negative for ERY974 staining are shown with “-.” Cyno means cynomolugus monkey.

We attempted to calculate NOAEL in cynomolgus monkeys. We first examined whether cynomolgus monkey is an appropriate species to determine the NOAEL of ERY974. In our previous study, we showed that the GPC3 arm and CD3 arm of ERY974 have a similar affinity between human and cynomolgus monkey counterparts using surface plasmon resonance analysis^10^. The equilibrium dissociation constant (*K*_D_) value of GPC3 in humans and cynomolgus monkeys was 1.46 × 10^–9^ mol/L (2.13 × 10^2^ ng/mL) and 1.35 × 10^–9^ mol/L (1.97 × 10^2^ ng/mL), respectively. The *K*_D_ value of CD3 in human and cynomolgus monkey was 2.07 × 10^–7^ mol/L (3.02 × 10^4^ ng/mL) and 1.55 × 10^–7^ mol/L (2.26 × 10^4^ ng/mL), respectively. Next, we examined the cytotoxicity of ERY974 using human- and cynomolgus monkey-derived PBMCs. In SK-pca13a cell line, in which moderate level of GPC3 was stably overexpressed in SK-HEP-1 with no GPC3 expression, the EC_50_ of cytotoxic activity presented only a few fold difference (human: 7.88 ng/mL, cynomolgus monkey: 16.06 ng/mL) (Fig. 4a,b). Similarly, the EC_50_ value of ERY974 to induce CD25 and CD69 expression in CD4^+^ T and CD8^+^ T cells was not much different between human and cynomolgus monkey within 4.82 fold difference (Supplementary Fig. 1a-e). Next, we performed tissue cross-reactivity study of ERY974 using normal tissue samples from humans and cynomolgus monkeys to examine whether ERY974-reactive tissues in human and cynomolgus monkey were common. First, we conducted IHC with ERY974 using xenograft tissues with different levels of GPC3 expression. As shown in Supplementary Fig. 2a, no staining was observed in the SK-HEP-1 xenograft tumor (no GPC3 expression). In contrast, ERY974 produced rare to occasional staining pattern in SK-pca31a xenograft tumor (low GPC3 expression) and weak to strong staining pattern in SK-pca13a xenograft tumor (moderate GPC3 expression) (Supplementary Fig. 2b, c). We confirmed that GPC3 expression level is one of the important factors to determine the sensitivity to ERY974 (Supplementary Fig. 2d). We then conducted IHC with ERY974 using normal tissues of human and cynomolgus monkey (Table S1). As depicted in Fig. 4c, we observed positive staining for ERY974 in mononuclear leukocytes in lymphoid tissue, peripheral blood, or other various tissues in which CD3^+^ cells are present. In both species, we also observed positive staining for ERY974 in trophoblast epithelium and decidual cells in the placenta (Supplementary Figs. 3a and 4a), endocrine cells in the pituitary (Supplementary Fig. 3b and 4b), and the epithelium in the thyroid (Supplementary Fig. 3c and 4c). Only in cynomolgus monkeys, positive staining for ERY974 was observed in the tubular epithelium in the kidney (Supplementary Fig. 4d) and theca cells and/or oocytes in the ovary (Supplementary Fig. 4e, f). These data show that cells or tissues which ERY974 binds to is mostly common between monkey and human. In kidney and ovary, ERY974 was detected only in cynomolgus monkey, which suggests that we may not underestimate the risk of safety in human from study results using cynomolgus monkey. Collectively, we concluded that cynomolgus monkey is an appropriate species to determine the NOAEL of ERY974.

### NOAEL of ERY974 was 0.1 μg/kg in the single-dose study in cynomolgus monkeys

We conducted a single-dose PK and toxicity study of ERY974 in cynomolgus monkey to determine the NOAEL. As shown in Table 1, three males and three females were administered with 0 and 10 μg/kg ERY974, and sacrificed on day 8 to examine an acute reaction, and the other three males and three females were administered with 0.1, 1 and 10 μg/kg ERY974, and sacrificed on day 22 to examine a recovery phase from an acute reaction. As summarized in Table 1, CRS-related clinical findings including red discolored skin (most frequently observed in the facial area), hyperthermia, and low-to-no food consumption were transiently observed in animals that were administered with 10 μg/kg ERY974, and supportive care, including administration of a non-steroidal anti-inflammatory drug, was provided to the animals. No cytokine release was observed at 0.1 μg/kg dose. At 1 μg/kg dose, a moderate increase of IL-6 was noted, and at 10 μg/kg dose, a marked, but transient, increase in IL-6 was observed as previously reported^10^. A slight increase in the IL-2, IL-5, and TNFα was observed at 10 μg/kg dose (Supplementary Fig. 5). Generally, ERY974-related clinical observations were resolved by day 6 post administration. At the interim sacrifice, pathological ERY974-related findings including decreased lymphocytes in the thymus, increased granulopoietic cells in the marrow of the femur and sternum, and increased infiltration of mononuclear cells and/or granulocytes in multiple tissues, were observed only at the 10 μg/kg dose (Table 1). However, the severity of these symptoms was minimal or slight. A decrease in the number of lymphocytes in the thymus correlated with decreased thymus weights, and this was commonly observed as an inflammatory response. At 1 μg/kg, cytokine release and associated general condition including red skin, increased body temperature, increased fibrinogen and triglyceride, decreased albumin in blood were observed, and therefore the NOAEL was determined as 0.1 μg/kg. We measured the area under the curve of plasma concentration versus time from 0 h (time zero) to Day 22 (AUC_all_),and maximum plasma concentration (C_max_) at 0.1 μg/kg as 4.00/3.32 ng·d/mL and 1.86/1.67 ng/mL, respectively.

**Table 1:** Summary of safety study of ERY974 in cynomolgus monkeys.

Group	Animals (n)	Autopsy (day)	Dose (μg/kg/dose)	Average maximum IL-6 level (pg/mL)^a^	Clinical signs/histopathological findings
1 (Control)	3 (M) + 3 (F)	8	0	48	No finding
2 (Low)	3 (M) + 3 (F)	22	0.1	32	No finding (NOAEL)
3 (Middle)	3 (M) + 3 (F)	22	1	231	Red skin, increased body temperature, increased fibrinogen and triglyceride, decreased albumin in blood,
4 (High)	3 (M) + 3 (F)	22	10	2505	Red skin on head and/or complete body, hyperthermia, decreased body weight, low-to-no food consumption, hunched posture, decreased reactivity, decreased lymphocytes in the thymus^b^, increased infiltration of mononuclear cells and/or granulocytes in multiple tissues, No evidence of damages in tissues (liver, kidney, or gastrointestinal)
5 (High)	3 (M) + 3 (F)	8	10		

The symptoms, level of cytokines in the blood, clinical pathological and histopathological findings in animals after administration of ERY974 are summarized. The level of IL-6 has been previously described^8^.

^a^8 h after administration.

^b^Observed on both day 8 and 22 autopsy samples.

*M* male, *F* female, *NOAEL* no observed adverse effect level.

### FIH dose was determined as 3 ng/kg based on the combination of the MABEL and NOAEL approaches

**Figure 5:**
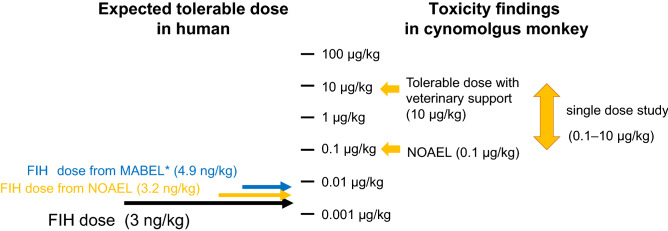
Calculation of the FIH dose from MABEL and NOAEL. The FIH dose from MABEL and NOAEL. The FIH dose from MABEL was calculated as the dose at which the C_max_ after 4 h of i.v. infusion of ERY974 is equal to the EC_10_ value of cytotoxicity assay, combining PK data, and the FIH dose from NOAEL was calculated considering species differences. FIH, first-in-human; MABEL, minimal anticipated biological effect level; NOAEL, observed adverse effect level.

As described in Fig. 5, the FIH dose from the MABEL approach, at which the predicted C_max_ after 4 h of intravenous (i.v) infusion of ERY974 was equal to the EC_10_ value in cytotoxic assay, was calculated as 4.9 ng/kg. Simultaneously, the FIH dose from NOAEL was calculated as 3.2 ng/kg taking a safety factor of 0.1 as a species difference into account^24^. By combining the MABEL and NOAEL approaches, we concluded that 3 ng/kg was the suitable FIH dose for the phase I clinical trial of ERY974.

## Discussion

As TGN1412 exerted severe adverse events in healthy volunteers who were administered the FIH dose, regulatory agencies have published a guideline for determining the FIH dose for immune-activating products^21,22,25^; the authorities suggested us to consider a MABEL approach when we determine FIH dose of immuno-oncology drugs. The FIH dose of 14 out of 17 T cell bispecific antibodies has been determined using the MABEL approach^26^. Therefore, accordingly, we applied MABEL to determine the FIH dose of ERY974 in addition to NOAEL. By combining these two methods, we determined 3 ng/kg as the FIH dose, which was slightly lower than the calculated dose of 4.9 ng/kg in the MABEL approach and 3.2 ng/kg in the NOAEL approach.

The FIH dose determined from 10% RO might be acceptable for conventional antibody drugs, but not for TRABs, as the FIH dose calculated from RO was mostly close to, and even beyond the maximum tolerance dose. Indeed, for ERY974, concentrations to achieve 10% RO for GPC3 and for CD3 were 24 and 3348 ng/mL, respectively, which were 300- and 40,000-fold higher than the EC_10_ obtained in the cytotoxicicty assay, respectively. It is considerably more complicated to interpret RO data of TRABs because the concentrations to achieve 10% RO would be different between those for CD3 and cancer antigen, and the proportions of T cells and cancer cells in tumors vary with the tumor microenvironment.

Previous literature demonstrated that the FIH dose calculated from murine efficacy dose is considerably different from the actual efficacy dose in patients as human immune system was not completely reconstituted in the murine model to evaluate TRABs. For example, in a preclinical model, the in vivo maximum efficacy dose of tarlatamab [a half-life extended (HLE) bispecific T-cell engager (BiTE) targeting DLL3] against patient-derived xenograft (PDX) tumor in a T cell-injected model using NOG mice was assessed to be 3 mg/kg^14^. However, in a phase 1 dose-escalation study, tarlatamab showed a partial response (PR) using a dose of 0.3 mg/patient^15^, which is approximately 4.3 μg/kg for a patient with a weight of 70 kg; the dose is several hundred lower than that determined in the preclinical model. This difference in efficacy dosage between mice and patients were also observed in AMG160, HLE PSMA-targeted BiTE. In a preclinical study, 0.2 mg/kg was required for a PR against 22Rv-1 tumor^16^. In contrast, in patients, 0.03 mg AMG160 dosage that corresponds to 0.4 μg/kg in a patient with a weight of 70 kg shows more than 50% reduction in prostate-specific antigen (PSA) level in blood^17,18^. These results suggest that murine models are not suitable to determine the FIH dose of TRABs. A method to determine the FIH dose of TRABs has been reported for carcinoembryonic antigen (CEA)-T cell bispecific and hEGFRvIII:CD3 bispecific single chain variable fragment, for which the MABEL approach was applied^27,28^. In both cases, the cytotoxicity assay was selected as the most sensitive assay. Cytotoxicity assay might be the most sensitive assay for various TRABs, including ERY974, regardless of their structural variability.

It was unexpected that huH-1 was the most sensitive cell line despite its slightly lower GPC3 expression than PC-10 and HepG2 as the efficacy of TRABs is normally dependent on antigen expression levels^10,14,29^. We confirmed that the efficacy of ERY974 is dependent on GPC3 expression level using SK-HEP-1 (no GPC3 expression), SK-pca31a (SK-HEP-1 expressing low level of GPC3), SK-pca13a (SK-HEP-1 expressing middle level of GPC3), and SK-pca60 (SK-HEP-1 expressing high level of GPC3) (Supplementary Fig. 2d). We assume that the GPC3 expression level in huH1 (6.6 × 10^4^) might be high enough to exert a maximum cytotoxicity of ERY974 as cytotoxicity is comparable between SK-pca13a (GPC3: 1.89 × 10^4^) and SK-pca60 (GPC3: 2.50 × 10^5^). Therefore, other factors probably have an influence on the sensitivity to TRABs in addition to the antigen expression level. Many literatures have been published so far on molecules that would be a determinant for sensitivity to cytotoxic lymphocyte (CTL)^30,31^. In common, signaling pathway of IFNγ and TNFα could be important to mediate cytotoxicity of CTL. We speculate that such pathway is more activated in huH1 than that in HepG2. However, we may still be able to consider hGPC3 expression level as one of the most important factors to predict the efficacy of ERY974.

In terms of a risk of toxicity, the application of EC_10_ value would be the conservative choice, as recently the use of EC_30_, even EC_50_, has been accepted by the FDA^26^. Unnecessarily low FIH doses could require numerous dose escalations to reach an efficacy dose, and therefore, many recruited patients might not be benefited. Hence, it is important to appropriately evaluate the balance of risk and benefit in patients. Strategies such as accelerated titration and/or intra-patient dose escalation could be also beneficial.

In conclusion, we determined the FIH dose of ERY974 as 3 ng/kg from both pharmacology and toxicology perspectives. In our phase I clinical trial (NCT02748837), we did not observe any adverse events in patient who were administered 3 ng/kg, demonstrating this approach as successful for ERY974^32^.

## Methods

### Ethics statement

The protocols of all animal studies were approved by the Institutional Animal Care and Use Committee (IACUC) of Chugai Pharmaceutical Co., Ltd. All animal studies were conducted in the animal facility accredited by the Association for Assessment and Accreditation of Laboratory Animal Care (AAALAC). All studies using human samples, e.g. PBMCs, were conducted according to the policy of the Chugai Ethical Committee. All studies were performed in accordance with relevant guidelines and regulations.

### Reagents

ERY974 was prepared by Chugai Pharmaceutical., Co., Ltd. (Tokyo, Japan).

### Cell lines

huH-1, a human hepatocellular carcinoma cell line, was purchased from Japanese Collection of Research Bioresources (JCRB)(Osaka, Japan). HepG2, a human hepatoblastoma cell line, and SK-HEP-1, a human liver cancer cell line, were purchased from American Type Culture Collection (ATCC)(Manassas, VA). SK-pca31a SK-pca13a, and SK-pca60 are derivatives of SK-HEP-1, to express GPC3 slightly, moderately, or strongly, respectively. PC-10, a human lung cancer cell line, was purchased from Immuno-Biological Laboratories (IBL)(Gunma, Japan). All cell lines were cultured according to the manufacturers’ instructions. We have not authenticated these cell lines by ourselves.

### PBMC preparation

All studies using human PBMCs were approved by the Chugai Ethical Committee, and study was conducted according to its policy. Informed consent was obtained from all donors through Chugai Pharmaceutical Co Ltd, or Cellular Technology Ltd. For various in vitro assays with huH-1, cryopreserved human frozen PBMCs from 10 different donors were purchased from Cellular Technology Ltd. (Shaker Heights, OH). For species comparison studies, human PBMCs were isolated from heparinized blood obtained from healthy volunteer donors by Ficoll–Paque PLUS (GE Healthcare, Chicago, IL) density gradient centrifugation, following the manufacturer’s instructions. Briefly, heparinized blood was diluted twice with PBS and transferred into a Leucosep (Greiner Bio-One, Frickenhausen, Germany). After centrifugation (2300 rpm, 10 min) at room temperature, PBMCs were washed twice with PBS (1100 rpm, 10 min) at room temperature, and the cells were counted. Cynomolgus monkey PBMCs were also isolated by Ficoll–Paque PLUS density gradient centrifugation without a Leucosep. After centrifugation (2300 rpm, 30 min) at room temperature, PBMCs were collected as described above.

### ABC of GPC3

The ABC of GPC3 in each cell line was determined using QIFIKIT (DAKO, Glostrup, Denmark) according to the manufacturer’s instructions. The cells were incubated with 20 μg/mL anti-GPC3 mouse mAb (in-house preparation) for 30 min at 4 °C. After washing, the cells were incubated with the secondary antibody (included in QIFIKIT) for 30 min at 4 °C. After further washing, the cells were analyzed using FACSLytic (BD, Franklin Lakes, NJ). The ABC was calculated using the calibration curve.

### Cytotoxicity assay

Cancer cells (1 × 10^4^/96 well), frozen PBMCs (2 × 10^5^/96 well), and ERY974 at various concentrations were mixed in a round-bottom 96 well plate and incubated for 24 h at 37 °C with 5% CO_2_. The lysed tumor cell-derived LDH was measured using the LDH Cytotoxicity Detection Kit (TAKARA Bio, Shiga, Japan). The cytotoxicity of ERY974 was calculated according to the manufacturer’s instructions.

### T cell activation assay

After the cytotoxicity assay, the remaining CD3^+^ cells were collected and used to measure the expression of CD69 and CD25. The cells were washed with 0.5 w/v % bovine serum albumin (BSA)/CellWASH (FACS/PBS) (BD, Franklin Lakes, NJ), and then diluted Fc blocking Reagent (Miltenyi, Gladbach, Germany) was added. The samples were incubated at room temperature for 10 min. Thereafter, antibodies against CD3 (APC-conjugated) (BD), CD25 (PE-conjugated) (BD), and CD69 (FITC-conjugated) (BD) were added, and the samples were incubated on ice for 30 min. After washing the cells with PBS, the samples were analyzed using FACSVerse (BD). Live/dead cell staining (Fixable Viability Dye eFluor 780; Thermo Fisher Scientific, Waltham, MA) was used to measure the live cell population. The data were analyzed using FlowJo software ver. 7.6.5 (Tomy Digital Biology) to calculate the CD25^+^ or CD69^+^ ratio in the CD3^+^ population.

### Measurement of cytokines

After performing the cytotoxicity assay described above, the remaining culture medium was collected and used to measure the levels of cytokines IL-2, IL-4, IL-6, IL-10, TNFα, and IFNγ using the CBA human Th1/Th2 cytokine kit II (BD) with FACSVerse. according to the manufacturer’s instructions. The data were analyzed using FCAP Array software ver. 3.0 (BD).

### Calculation of RO

RO was calculated using the following formula:

$${\text{RO}}\;\left( \% \right) = \left[ {{\text{mAb}}} \right]{/}\left( {\left[ {K_{{\text{D}}} } \right] + \left[ {{\text{mAb}}} \right]} \right) \times 100.$$

The *K*_D_ values of ERY974 to GPC3 and CD3ε measured by Surface i Resonance in which various concentrations of ERY974 was applied on the immobilized GPC3 and/or CD3 ε peptide was were 1.46 × 10^–9^ and 2.07 × 10^–7^ mol/L, respectively^8^.

### Tissue cross-reactivity study

All studies were conducted in Charles River Laboratories, Inc. (Frederick, MD) according to the policy of the IACUC. All studies using human samples were conducted according to the policy of the Chugai Ethical Committee, and informed consent was obtained for all human-derived samples through each company, or institute described as follows. Tissue samples were collected as surgical or autopsy specimens from humans, obtained from Charles River Laboratories, Cooperative Human Tissue Network (Nashville, TN), National Disease Research Interchange (Philadelphia, PA), Cureline (Brisbane, CA), Poietics (Basel, Switzerland), Analytical Biological Services (Wilmington, DE), Frederick Memorial Hospital (Frederick, MD), or Nationwide Children’s Hospital (Columbus, OH), and as necropsy specimens from cynomolgus monkeys, obtained from Charles River Laboratories, Covance (Burlington, NC), Bioqual, Inc. (Rockville, MD), or Alpha Genesis (Yemassee, SC), filled with Tissue-Tek OCT Compound (Sakura Finetek, Street Torrance, CA), and stored in a freezer at − 85 °C to − 70 °C. The tissues were cut into approximately 5 μm-thick sections and fixed in acetone. As for control samples, NOD/ShiJic-scidJcl (NOD/SCID) mice aged 5 weeks were purchased from CLEA Japan, Inc (Tokyo, Japan). After an acclimatization of 1~3 weeks, 1 × 10^7^ cells of SK-HEP-1, SK-pca31a, and SK-pca13a were subcutaneously inoculated into the right flank of mice. Established tumor was taken and filled with Tek OCT Compound. For immunohistochemistry (IHC), modified methods of Tuson et al., Fung et al., and Hierck et al. were used^33–35^. Briefly, ERY974 or isotype control antibody was mixed with biotinylated F(ab′)2 donkey anti-human IgG, Fcγ fragment-specific antibody (DkαHuIgG) (Jackson ImmunoResearch Laboratories, West Grove, PA), and incubated overnight at 4 °C. The slides were then treated with the ABC Elite reagent (Vector Laboratories, Burlingame CA) for 30 min, rinsed with TBS, and then treated with DAB (Sigma-Aldrich, St. Louis, MO) for 4 min as a substrate for peroxidase.

### Single-dose study in cynomolgus monkeys

The study in cynomolgus monkeys (*Macaca fascicularis*) was conducted in Covance Inc. according to ARRIVE (Animal Research: Reporting of In Vivo Experiments) guidelines^36^. All procedures were approved by the IACUC, and the facility was accredited by the AAALAC. Male and female cynomolgus monkeys were assigned to five groups; each group consisted of three males and three females. The monkeys were dosed once with ERY974 or with the vehicle via i.v infusion via a saphenous vein for approximately 30 min on day 1. The dose levels were 0, 0.1, 1, 10, and 10 μg/kg for Groups 1, 2, 3, 4, and 5, respectively. The animals in Groups 1 and 5 were sacrificed on day 8 of the dosing phase, whereas those in Groups 2, 3, and 4 were sacrificed on day 22 of the dosing phase. Blood samples were collected via the femoral vein in the pre-dose phase and in the dosing phase at approximately 2, 8, 24, 48, 72, and 168 h for cytokine measurement using a CBA non-human primate Th1/Th2 cytokine kit (BD). On day 8 of the dosing phase, all animals in Groups 1 and 5 were fasted overnight, anesthetized with sodium pentobarbital, exsanguinated, and necropsied. On day 22 of the dosing phase, all animals in Groups 2, 3, and 4 were treated as described above. Most of the tissues from each animal were preserved in 10% neutral-buffered formalin, and embedded in paraffin, sectioned, sliced, and stained with hematoxylin and eosin for macroscopic examinations.

### FIH dose calculation based on the MABEL and NOAEL approaches

In the MABEL approach, the FIH dose, which corresponds to the plasma concentration in patients after 4 h of i.v. infusion of ERY974 and to the EC_10_ value obtained in the cytotoxicity assay (0.0767 ng/mL), was determined. human PK profile was predicted using an allometric scaling method in cynomolgus monkeys. Furthermore, a conventional NOAEL-based approach using toxicity data in cynomolgus monkeys was also adopted to calculate the FIH dose using body surface area correction, considering the differences between species.

## Supplementary Information

Supplementary Information.

## Data Availability

Data would be available by reasonable request from the corresponding author.
