# Supplementary material for: Determination of starting dose of the T cell-redirecting bispecific antibody ERY974 targeting glypican-3 in first-in-human clinical trial
Source: Sci Rep. 2022 Jul 19;12:12312. doi: 10.1038/s41598-022-16564-x (PMC9296674; doi:10.1038/s41598-022-16564-x)
Supplement: Supplementary file 1 — Supplementary Information. [file 41598_2022_16564_MOESM1_ESM.pdf]

Supplementary information

Determination of starting dose of the T cell-redirecting bispecific antibody ERY974 targeting glypican-3 in first-in-human clinical trial

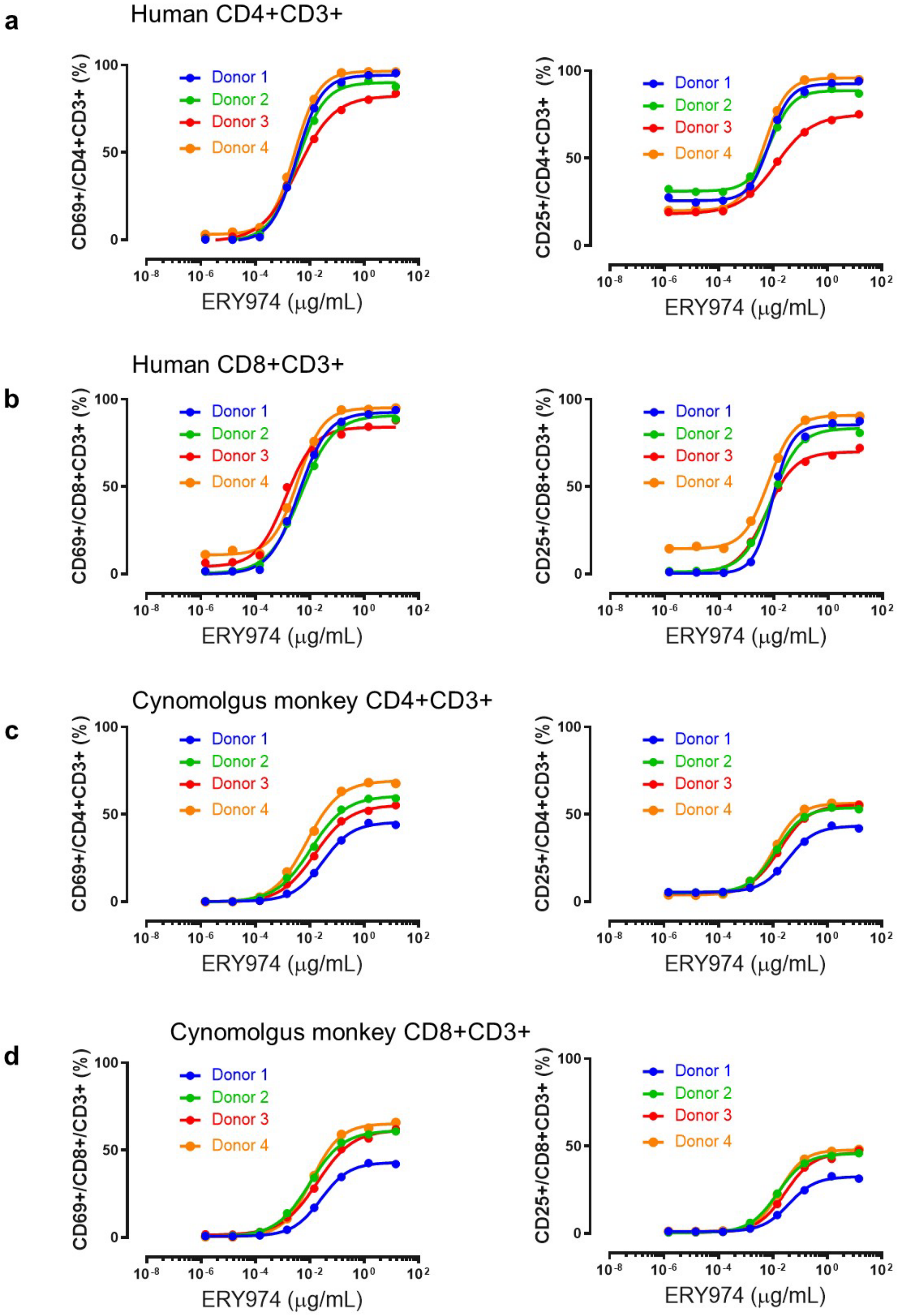

e

EC50 (μg/mL)	Human		Cynomolgus monkey	
	CD4+	CD8+	CD4+	CD8+
CD25+	0.00851	0.00733	0.0197	0.0250
CD69+	0.00336	0.00345	0.0162	0.0166

Supplementary Figure 1. T cell activation assay with ERY974 using human and cynomolgus monkey PBMCs. a, Human $CD4^+$ T cell activation induced by ERY974. **b**, Human $CD8^+$ T cell activation induced by ERY974. **c**, Cynomolgus monkey $CD4^+$ T cell activation induced by ERY974. **d**, Cynomolgus monkey $CD8^+$ T cell activation induced by ERY974. T cell activation by ERY974 was measured using cytometric analysis for T cell activation markers CD25 and CD69. PBMCs from four independent donors were used as effector cells, and SK-pca13a, that was engineered to express GPC3 in SK-HEP-1 in which GPC3 is not expressed, was used as the target cell. Data were obtained with n = 1, **e**, EC_{50} values of T cell activation assay in **a**-**d**.

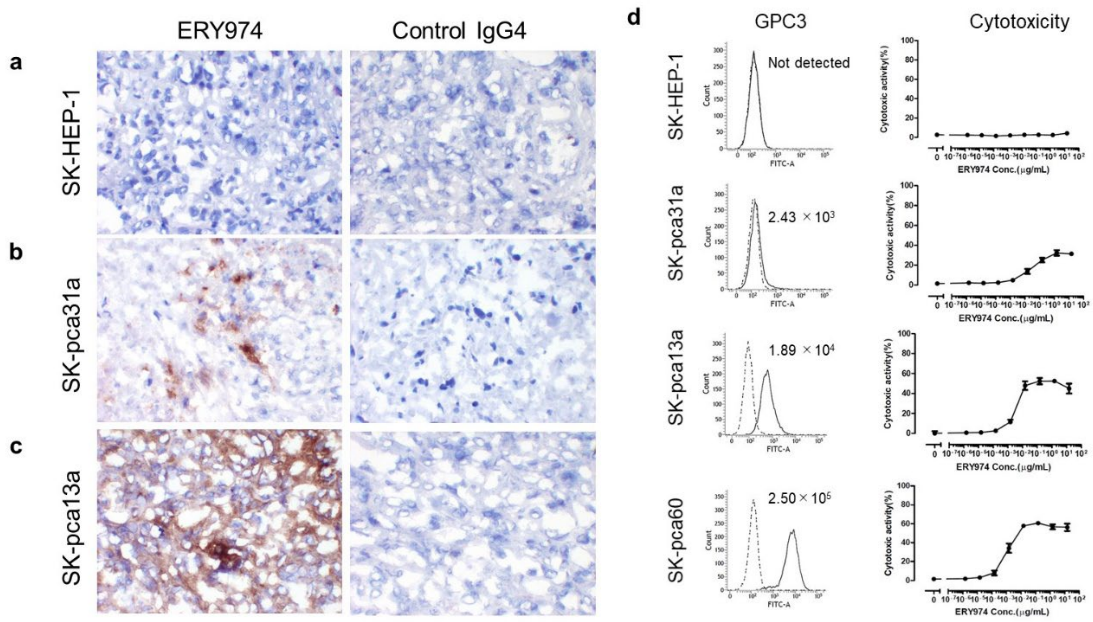

Supplementary Figure 2. Cryosections of SK-HEP-1, SK-pca31a, and SK-pca13a -xenograft tumor tissues stained with ERY974, and cytotoxicity of ERY974 in these cells. a, Cryosections of SK-HEP-1, GPC3^{-} xenograft tumor (left: stained with ERY974, right: stained with control IgG4). **b**, Cryosections of SK-pca31a, GPC3-low tumor (left: stained with ERY974, right: stained with control IgG4). **c**, Cryosections of SK-pca13a, GPC3-moderate tumor (left: stained with ERY974, right: stained with control IgG4). SK-HEP-1 was engineered to express exogenous GPC3. GPC3 is expressed in SK-pca31a and SK-pca13a at low and medium level, respectively. **d**, Correlation of GPC3 expression level with cytotoxicity of ERY974 in SK-HEP-1, SK-pca31a, SK-pca13a, and SK-pca60. SK-pca60 was an engineered cell to strongly express exogenous GPC3 in SK-HEP-1. The ABC of GPC3 determined using QIFI kit

in SK-pca31a, SK-pca13a, and SK-pca60 is 2.43×10^3, 1.89×10^4, and 2.50×10^5, respectively. Cytotoxicity of ERY974 was examined using LDH release assay. Data are shown as mean ± SD (n = 3).

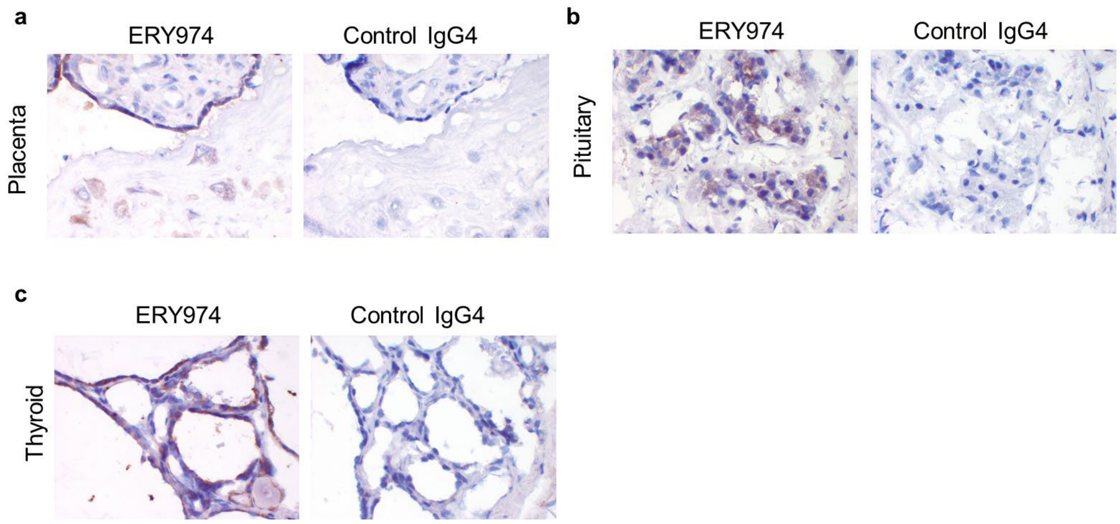

Supplementary Figure 3. Cryosections of human normal tissues stained with ERY974. Cryosections of human (**a**) placenta, (**b**) pituitary, and (**c**) thyroid stained with ERY974. Immunohistochemistry was performed with tissues from three different individuals. Representative images are shown. The left images show the staining with ERY974, and the right ones show the staining with control IgG4.

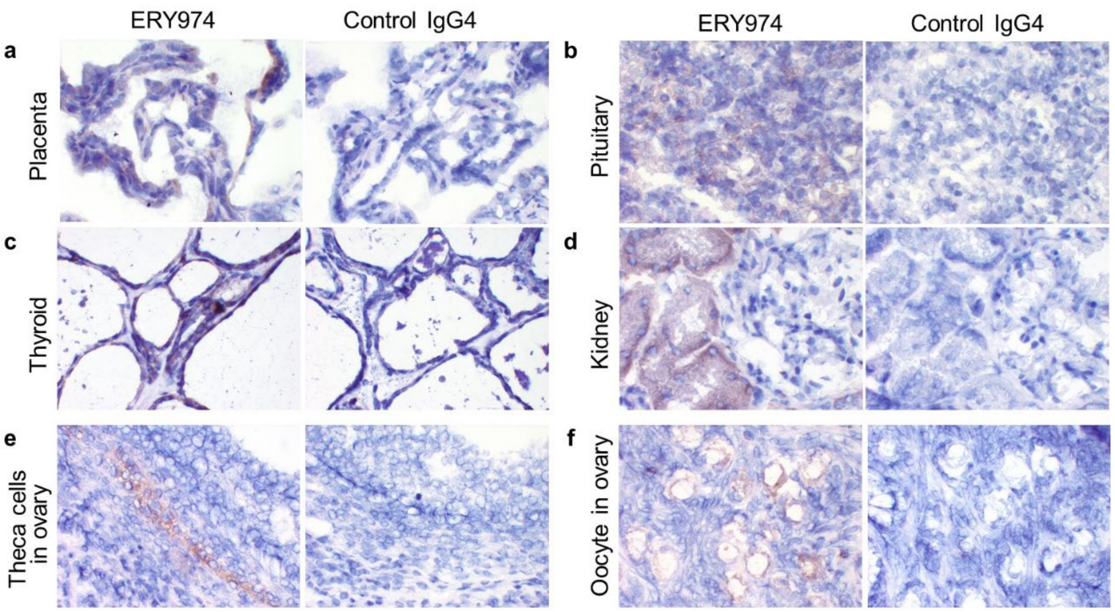

Supplementary Figure 4. Cryosections of cynomolgus monkey normal tissues stained with ERY974. Cryosections of cynomolgus monkey (**a**) placenta, (**b**) pituitary, (**c**) thyroid, (**d**) kidney, (**e**) theca cells in ovary, and (**f**) oocytes in ovary stained with ERY974. Immunohistochemistry was performed using tissues from three different individuals. Representative images are shown. The left images show the staining with ERY974, and the right ones show the staining with control IgG4.

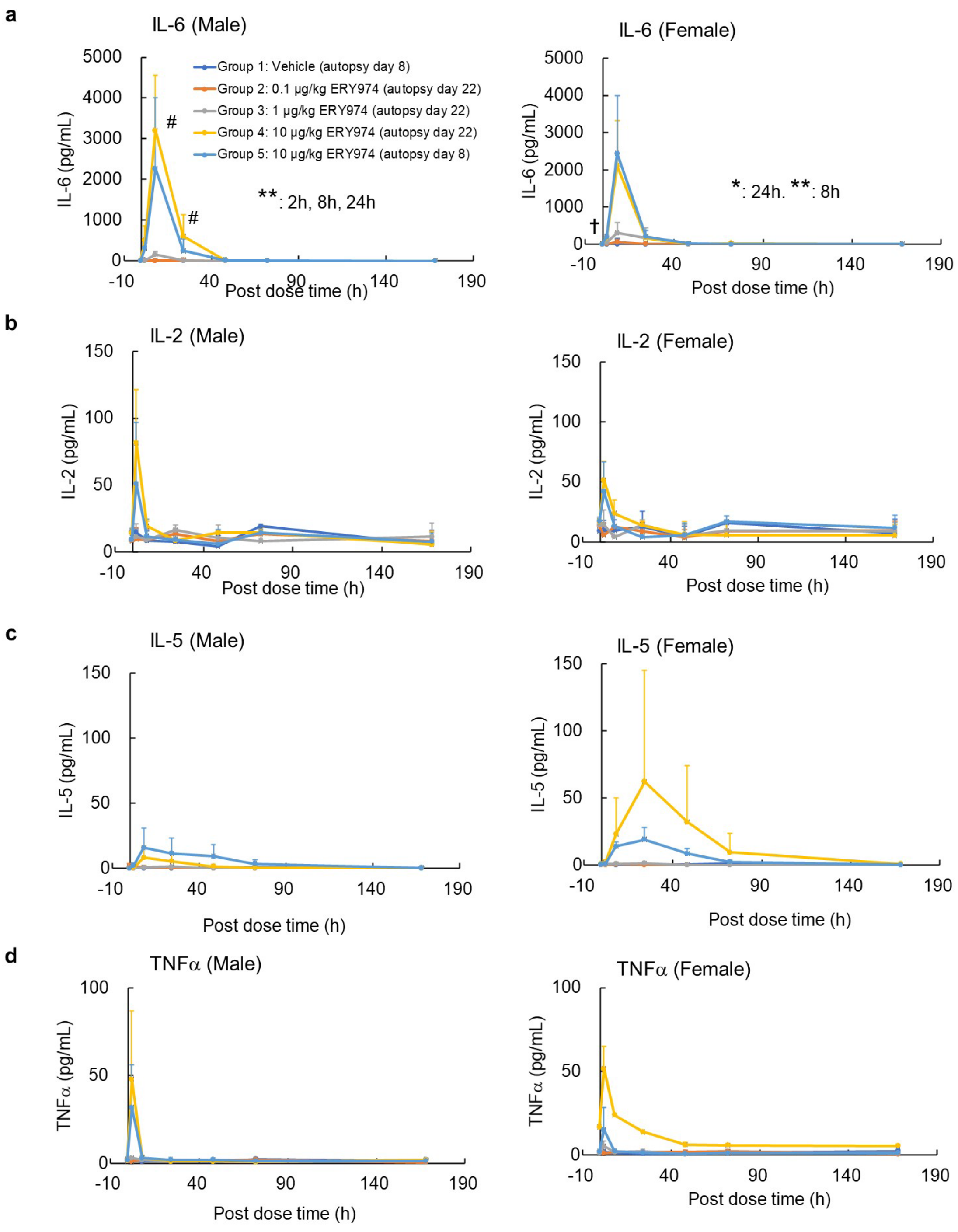

Supplementary Figure 5. Serum cytokine level in cynomolgus monkeys after the administration of ERY974 in the single dose study. a, Serum IL-6 level after the administration of ERY974 in the Good Laboratory Practice (GLP)-compliant single

dose toxicity study (left: male, right: female). **b**, Serum IL-2 level after the administration of ERY974 in the single dose study (left: male, right: female). **c**, Serum IL-5 level after the administration of ERY974 in the single dose study (left: male, right: female). **d**, Serum TNFα level after the administration of ERY974 in the single dose study (left: male, right: female). Data are shown as mean ± SD (n = 3). The extrapolated values below the lower limit of quantification (LLOQ: 20 pg/mL) were used as reference data. Data was analyzed for homogeneity of variance by Bartlett's test. When the variance was homogeneous, Dunnett's test was performed for multiple comparison between the control group and each test article group. When the variance was heterogeneous by Bartlett's test, a Dunnett-type test (Miller's test) was performed for multiple comparison between the control group and each test article group. SAS System (Release 9.2, SAS Institute Inc.) was used for these statistical analyses at a significance level of 5% for Bartlett's test or at a two-sided significance level of 5% for other tests. *P < 0.05, **P < 0.01 with Bartlett's test, # P < 0.05 with Miller's test, and † P < 0.01 with Dunnett's test. No statistical significance was observed between vehicle and test groups in all cytokines except IL-6.

Supplementary Table 1. Human and cynomolgus monkey tissues used in the tissue cross-reactivity study.

Adrenal	Heart	Salivary gland
Bladder (urinary)	Kidney (glomerulus, tubule)	Skin
Blood cells[a]	Liver	Spinal cord
Blood vessels (endothelium)[b]	Lung	Spleen
Bone marrow	Lymph node	Striated muscle (skeletal)
Brain-cerebellum	Ovary	Testis
Brain-cerebellum (cerebral cortex)	Pancreas	Thymus
Breast	Parathyroid	Thyroid
Colon (large intestine)	Peripheral nerve	Tonsil
Eye	Pituitary	Ureter
Fallopian tube	Placenta	Uterus-cervix
Gastrointestinal tract[c]	Prostate	Uterus-endometrium

Tissue samples were collected as surgical autopsy specimens from human subjects and as necropsy specimens from cynomolgus monkeys.

[a]Evaluated from peripheral blood smears

[b]Evaluated from all tissues where present

[c]Includes the esophagus, small intestine, and stomach (including underlying smooth muscle)
